# (*E*)-1-(3-Nitro­phen­yl)ethanone (2-methyl­phen­yl)hydrazone

**DOI:** 10.1107/S1600536811037846

**Published:** 2011-09-30

**Authors:** A. Zeb, S. Yousuf

**Affiliations:** a H.E.J. Research Institute of Chemistry, International Center for Chemical and Biological Sciences, University of Karachi, Karachi 75270, Pakistan

## Abstract

In the title Schiff base compound, C_15_H_15_N_3_O_2_, the azomethine double bond adopts an *E* configuration. The dihedral angle between the two aromatic rings is 13.4 (12)°. In the crystal, mol­ecules are arranged in wave-like layers parallel to (100) without any classical hydrogen bonding.

## Related literature

For the biological activit of Schiff bases, see: Khan *et al.* (2009 ▶); Gerdemann *et al.* (2002 ▶); Mallikarjun & Sangamesh (1997 ▶); Solomon & Lowery (1993 ▶). For the role of Schiff bases and Amadori products in the process of glycation, see: Ahmad *et al.* (2007 ▶); Ahmed (2005) ▶. For the crystal structures of closely related compounds see: Fun *et al.* (2008 ▶); Tezcan *et al.* (2004 ▶).
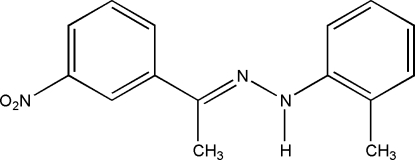


## Experimental

### 

#### Crystal data

C_15_H_15_N_3_O_2_
*M* *_r_* = 269.30
Monoclinic, 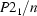

*a* = 7.4763 (18) Å
*b* = 25.742 (6) Å
*c* = 7.6564 (19) Å
β = 110.485 (5)°
*V* = 1380.3 (6) Å^3^
*Z* = 4
Mo *K*α radiation
μ = 0.09 mm^−1^
*T* = 273 K
0.51 × 0.46 × 0.08 mm

#### Data collection

Bruker SMART APEX CCD area-detector diffractometer
Absorption correction: multi-scan (*SADABS*; Bruker, 2000 ▶) *T*_min_ = 0.956, *T*_max_ = 0.993
7958 measured reflections
2535 independent reflections
1746 reflections with *I* > 2σ(*I*)
*R*_int_ = 0.039

#### Refinement

*R*[*F* ^2^ > 2σ(*F* ^2^)] = 0.060
*wR*(*F* ^2^) = 0.176
*S* = 1.04
2535 reflections
187 parameters
H atoms treated by a mixture of independent and constrained refinement
Δρ_max_ = 0.29 e Å^−3^
Δρ_min_ = −0.21 e Å^−3^

### 

Data collection: *SMART* (Bruker, 2000 ▶); cell refinement: *SAINT* (Bruker, 2000 ▶); data reduction: *SAINT*; program(s) used to solve structure: *SHELXS97* (Sheldrick, 2008 ▶); program(s) used to refine structure: *SHELXL97* (Sheldrick, 2008 ▶); molecular graphics: *SHELXTL* (Sheldrick, 2008 ▶); software used to prepare material for publication: *SHELXTL*, *PARST* (Nardelli, 1995 ▶) and *PLATON* (Spek, 2009 ▶).

## Supplementary Material

Crystal structure: contains datablock(s) global, I. DOI: 10.1107/S1600536811037846/rz2637sup1.cif

Structure factors: contains datablock(s) I. DOI: 10.1107/S1600536811037846/rz2637Isup2.hkl

Supplementary material file. DOI: 10.1107/S1600536811037846/rz2637Isup3.cml

Additional supplementary materials:  crystallographic information; 3D view; checkCIF report

## Figures and Tables

**Fig. 1.**
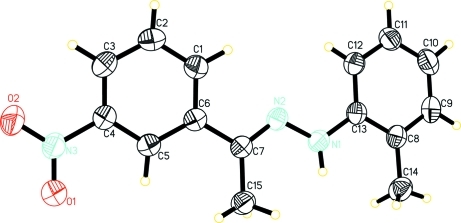
The molecular structure of the title compound with displacement ellipsoids drawn at 30% probability level.

**Fig. 2.**
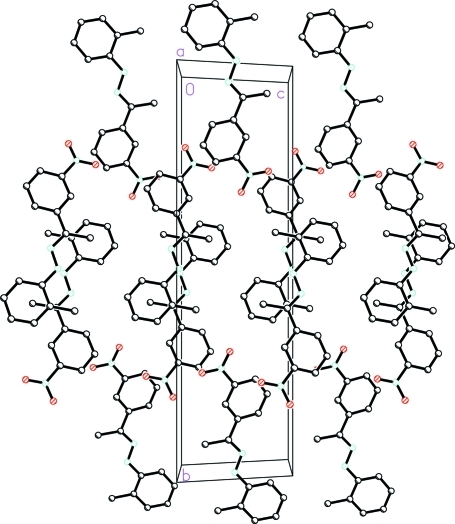
The crystal packing of the title compound viewed down the a axis. Hydrogen atoms are omitted.

## supplementary crystallographic information

### Comment

Schiff bases represent an important class of organic compounds, with a wide range of biological properties including antifungal, antibacterial, herbicidal, antiproliferative, cytotoxic, anticonvulsant and anticancer activities (Khan *et al.*, 2009; Gerdemann *et al.*, 2002; Mallikarjun & Sangamesh, 1997; Solomon & Lowery, 1993). They are also known as important intermediates formed during the process of glycation (reaction of protein and glucose) undergoing rearrangement to form more stable Amadori products, which are considered as therapeutic targets to treat diabetes and its complications (Ahmad *et al.*, 2007; Ahmed, 2005). During our on going search for effective antiglycating agents, the title compound was prepared and crystallized.

The structure of title compound (Fig. 1) is not planar, the dihedral angle between the aromatic rings (C1—C6 and C8—C13) being 13.4 (12)°. The azomethine (C7═N2) double bond adopts an *E* configuration, with the N1–N2–C7—C6 torsion angle of 178.38 (16)°. The bond lengths and angle are similar to those observed in other structurally related compounds (Fun *et al.*, 2008; Tezcan *et al.*, 2004). In the crystal structure, the molecules are arranged in wave-like layers parallel to the (100) plane (Fig. 2) without any classical intermolecular hydrogen bonding.

### Experimental

The synthesis of title compound I was carried out by refluxing a mixture of 3-nitroacetophenone (165 mg, 1 mmol) and 1-(2-methylphenyl)hydrazine hydrochloride (159 mg, 1 mmol) with acetic acid (1 ml) in ethanol (10 ml) for 18 h. The progress of reaction was monitored by TLC. After cooling and filtration the crystalline product was collected, washed with hexane and dried to afford the title compound in 85% yield. Recrystallization from ethanol afforded yellowish crystals suitable for single-crystal X-ray diffraction studies. All chemicals were purchased from Sigma-Aldrich.

### Refinement

H atoms on methyl and methine groups were positioned geometrically with C—H = 0.96 and 0.93 Å respectively, and constrained to ride on their parent atoms with *U*_iso_(H) = 1.2*U*_eq_(C) or 1.5*U*_eq_(C) for methyl H atoms. The hydrazone H atom was located in a difference Fourier map and refined isotropically. A rotating group model was applied to the methyl groups.

### Crystal data

**Table:**

C_15_H_15_N_3_O_2_	*F*(000) = 568
*M**_r_* = 269.30	*D*_x_ = 1.296 Mg m^−^^3^
Monoclinic, *P*2_1_/*n*	Mo *K*α radiation, λ = 0.71073 Å
Hall symbol: -P 2yn	Cell parameters from 2070 reflections
*a* = 7.4763 (18) Å	θ = 3.0–24.4°
*b* = 25.742 (6) Å	µ = 0.09 mm^−^^1^
*c* = 7.6564 (19) Å	*T* = 273 K
β = 110.485 (5)°	Plate, yellow
*V* = 1380.3 (6) Å^3^	0.51 × 0.46 × 0.08 mm
*Z* = 4	

### Data collection

**Table:**

Bruker SMART APEX CCD area-detector diffractometer	2535 independent reflections
Radiation source: fine-focus sealed tube	1746 reflections with *I* > 2σ(*I*)
graphite	*R*_int_ = 0.039
ω scans	θ_max_ = 25.5°, θ_min_ = 1.6°
Absorption correction: multi-scan (*SADABS*; Bruker, 2000)	*h* = −9→9
*T*_min_ = 0.956, *T*_max_ = 0.993	*k* = −31→29
7958 measured reflections	*l* = −9→9

### Refinement

**Table:**

Refinement on *F*^2^	Primary atom site location: structure-invariant direct methods
Least-squares matrix: full	Secondary atom site location: difference Fourier map
*R*[*F*^2^ > 2σ(*F*^2^)] = 0.060	Hydrogen site location: inferred from neighbouring sites
*wR*(*F*^2^) = 0.176	H atoms treated by a mixture of independent and constrained refinement
*S* = 1.04	*w* = 1/[σ^2^(*F*_o_^2^) + (0.0991*P*)^2^ + 0.0824*P*] where *P* = (*F*_o_^2^ + 2*F*_c_^2^)/3
2535 reflections	(Δ/σ)_max_ < 0.001
187 parameters	Δρ_max_ = 0.29 e Å^−^^3^
0 restraints	Δρ_min_ = −0.21 e Å^−^^3^

### Special details

**Table:**

Geometry. All e.s.d.'s (except the e.s.d. in the dihedral angle between two l.s. planes) are estimated using the full covariance matrix. The cell e.s.d.'s are taken into account individually in the estimation of e.s.d.'s in distances, angles and torsion angles; correlations between e.s.d.'s in cell parameters are only used when they are defined by crystal symmetry. An approximate (isotropic) treatment of cell e.s.d.'s is used for estimating e.s.d.'s involving l.s. planes.
Refinement. Refinement of *F*^2^ against ALL reflections. The weighted *R*-factor *wR* and goodness of fit *S* are based on *F*^2^, conventional *R*-factors *R* are based on *F*, with *F* set to zero for negative *F*^2^. The threshold expression of *F*^2^ > σ(*F*^2^) is used only for calculating *R*-factors(gt) *etc*. and is not relevant to the choice of reflections for refinement. *R*-factors based on *F*^2^ are statistically about twice as large as those based on *F*, and *R*- factors based on ALL data will be even larger.

### Fractional atomic coordinates and isotropic or equivalent isotropic displacement parameters (Å^2^)

**Table:**

	*x*	*y*	*z*	*U*_iso_*/*U*_eq_	
O1	−0.0105 (4)	0.25761 (8)	0.7851 (3)	0.1120 (8)	
O2	−0.0998 (3)	0.30826 (7)	0.5495 (3)	0.0983 (6)	
N1	0.2485 (3)	0.00558 (7)	0.5057 (3)	0.0652 (5)	
N2	0.1857 (2)	0.05385 (7)	0.4420 (2)	0.0600 (5)	
N3	−0.0373 (3)	0.26679 (8)	0.6232 (3)	0.0753 (6)	
C1	0.0995 (4)	0.15320 (9)	0.3011 (3)	0.0716 (6)	
H1B	0.1320	0.1284	0.2290	0.086*	
C2	0.0380 (4)	0.20121 (10)	0.2255 (3)	0.0780 (7)	
H2B	0.0275	0.2082	0.1032	0.094*	
C3	−0.0082 (3)	0.23888 (9)	0.3293 (3)	0.0705 (6)	
H3A	−0.0495	0.2716	0.2797	0.085*	
C4	0.0086 (3)	0.22673 (8)	0.5084 (3)	0.0603 (5)	
C5	0.0684 (3)	0.17883 (8)	0.5874 (3)	0.0581 (5)	
H5A	0.0778	0.1722	0.7097	0.070*	
C6	0.1147 (3)	0.14059 (8)	0.4826 (3)	0.0557 (5)	
C7	0.1796 (3)	0.08832 (8)	0.5615 (3)	0.0548 (5)	
C8	0.3256 (3)	−0.08197 (8)	0.4538 (3)	0.0619 (6)	
C9	0.3307 (3)	−0.12021 (9)	0.3286 (4)	0.0759 (7)	
H9A	0.3731	−0.1533	0.3731	0.091*	
C10	0.2748 (4)	−0.11080 (11)	0.1401 (4)	0.0856 (8)	
H10A	0.2791	−0.1372	0.0588	0.103*	
C11	0.2130 (4)	−0.06233 (11)	0.0734 (3)	0.0821 (7)	
H11A	0.1747	−0.0558	−0.0539	0.099*	
C12	0.2068 (3)	−0.02331 (9)	0.1923 (3)	0.0691 (6)	
H12A	0.1665	0.0097	0.1456	0.083*	
C13	0.2604 (3)	−0.03265 (8)	0.3823 (3)	0.0576 (5)	
C14	0.3872 (4)	−0.09290 (10)	0.6573 (3)	0.0836 (7)	
H14A	0.4376	−0.1275	0.6814	0.125*	
H14B	0.2796	−0.0898	0.6976	0.125*	
H14C	0.4840	−0.0685	0.7242	0.125*	
C15	0.2345 (4)	0.07845 (9)	0.7658 (3)	0.0728 (6)	
H15A	0.3563	0.0614	0.8110	0.109*	
H15B	0.1401	0.0567	0.7877	0.109*	
H15C	0.2422	0.1109	0.8299	0.109*	
H1N1	0.285 (4)	−0.0027 (9)	0.622 (4)	0.079 (8)*	

### Atomic displacement parameters (Å^2^)

**Table:**

	*U*^11^	*U*^22^	*U*^33^	*U*^12^	*U*^13^	*U*^23^
O1	0.164 (2)	0.0973 (14)	0.0794 (12)	0.0453 (13)	0.0483 (12)	0.0098 (10)
O2	0.1248 (17)	0.0622 (11)	0.0986 (13)	0.0146 (10)	0.0274 (11)	0.0023 (9)
N1	0.0681 (13)	0.0615 (11)	0.0611 (11)	0.0066 (9)	0.0163 (9)	0.0031 (9)
N2	0.0516 (11)	0.0601 (11)	0.0647 (10)	0.0009 (8)	0.0160 (8)	0.0015 (8)
N3	0.0795 (14)	0.0627 (12)	0.0776 (13)	0.0076 (10)	0.0199 (10)	0.0030 (10)
C1	0.0782 (16)	0.0720 (15)	0.0702 (14)	0.0032 (11)	0.0332 (12)	0.0025 (11)
C2	0.0897 (19)	0.0802 (17)	0.0677 (14)	0.0014 (13)	0.0320 (13)	0.0129 (12)
C3	0.0662 (15)	0.0631 (14)	0.0774 (15)	−0.0015 (11)	0.0192 (11)	0.0118 (11)
C4	0.0520 (13)	0.0586 (12)	0.0665 (12)	−0.0038 (9)	0.0161 (10)	0.0011 (10)
C5	0.0509 (12)	0.0613 (13)	0.0574 (11)	−0.0066 (9)	0.0130 (9)	0.0002 (9)
C6	0.0431 (12)	0.0613 (12)	0.0592 (11)	−0.0076 (9)	0.0134 (9)	−0.0007 (9)
C7	0.0432 (11)	0.0590 (12)	0.0593 (11)	−0.0054 (9)	0.0144 (9)	−0.0009 (9)
C8	0.0457 (12)	0.0651 (13)	0.0742 (13)	−0.0017 (9)	0.0200 (10)	0.0006 (10)
C9	0.0613 (15)	0.0673 (15)	0.1014 (18)	0.0070 (11)	0.0315 (13)	−0.0033 (12)
C10	0.0767 (18)	0.095 (2)	0.0891 (18)	0.0090 (14)	0.0334 (14)	−0.0222 (14)
C11	0.0724 (17)	0.106 (2)	0.0678 (14)	0.0161 (14)	0.0244 (12)	−0.0065 (14)
C12	0.0586 (14)	0.0786 (15)	0.0685 (13)	0.0098 (11)	0.0202 (11)	0.0034 (11)
C13	0.0413 (11)	0.0634 (13)	0.0673 (13)	−0.0017 (9)	0.0180 (9)	−0.0010 (10)
C14	0.0877 (19)	0.0762 (16)	0.0867 (17)	0.0121 (13)	0.0302 (14)	0.0189 (13)
C15	0.0769 (17)	0.0654 (14)	0.0697 (14)	0.0015 (11)	0.0180 (12)	0.0036 (10)

### Geometric parameters (Å, °)

**Table:**

O1—N3	1.207 (2)	C7—C15	1.493 (3)
O2—N3	1.222 (2)	C8—C9	1.384 (3)
N1—N2	1.357 (2)	C8—C13	1.401 (3)
N1—C13	1.389 (3)	C8—C14	1.489 (3)
N1—H1N1	0.86 (2)	C9—C10	1.376 (3)
N2—C7	1.286 (2)	C9—H9A	0.9300
N3—C4	1.471 (3)	C10—C11	1.366 (4)
C1—C2	1.374 (3)	C10—H10A	0.9300
C1—C6	1.392 (3)	C11—C12	1.367 (3)
C1—H1B	0.9300	C11—H11A	0.9300
C2—C3	1.372 (3)	C12—C13	1.388 (3)
C2—H2B	0.9300	C12—H12A	0.9300
C3—C4	1.369 (3)	C14—H14A	0.9600
C3—H3A	0.9300	C14—H14B	0.9600
C4—C5	1.377 (3)	C14—H14C	0.9600
C5—C6	1.388 (3)	C15—H15A	0.9600
C5—H5A	0.9300	C15—H15B	0.9600
C6—C7	1.485 (3)	C15—H15C	0.9600
			
N2—N1—C13	120.06 (18)	C9—C8—C14	121.1 (2)
N2—N1—H1N1	122.9 (16)	C13—C8—C14	121.22 (19)
C13—N1—H1N1	117.0 (16)	C10—C9—C8	122.0 (2)
C7—N2—N1	118.04 (17)	C10—C9—H9A	119.0
O1—N3—O2	123.0 (2)	C8—C9—H9A	119.0
O1—N3—C4	119.10 (19)	C11—C10—C9	119.4 (2)
O2—N3—C4	117.9 (2)	C11—C10—H10A	120.3
C2—C1—C6	121.9 (2)	C9—C10—H10A	120.3
C2—C1—H1B	119.1	C10—C11—C12	120.6 (2)
C6—C1—H1B	119.1	C10—C11—H11A	119.7
C3—C2—C1	120.5 (2)	C12—C11—H11A	119.7
C3—C2—H2B	119.8	C11—C12—C13	120.4 (2)
C1—C2—H2B	119.8	C11—C12—H12A	119.8
C4—C3—C2	117.7 (2)	C13—C12—H12A	119.8
C4—C3—H3A	121.1	C12—C13—N1	121.79 (19)
C2—C3—H3A	121.1	C12—C13—C8	120.01 (19)
C3—C4—C5	123.11 (19)	N1—C13—C8	118.19 (19)
C3—C4—N3	118.66 (19)	C8—C14—H14A	109.5
C5—C4—N3	118.24 (19)	C8—C14—H14B	109.5
C4—C5—C6	119.32 (19)	H14A—C14—H14B	109.5
C4—C5—H5A	120.3	C8—C14—H14C	109.5
C6—C5—H5A	120.3	H14A—C14—H14C	109.5
C5—C6—C1	117.51 (19)	H14B—C14—H14C	109.5
C5—C6—C7	121.31 (18)	C7—C15—H15A	109.5
C1—C6—C7	121.17 (18)	C7—C15—H15B	109.5
N2—C7—C6	115.08 (17)	H15A—C15—H15B	109.5
N2—C7—C15	124.21 (18)	C7—C15—H15C	109.5
C6—C7—C15	120.72 (17)	H15A—C15—H15C	109.5
C9—C8—C13	117.6 (2)	H15B—C15—H15C	109.5
			
C13—N1—N2—C7	−178.96 (16)	C5—C6—C7—N2	168.33 (17)
C6—C1—C2—C3	1.0 (4)	C1—C6—C7—N2	−12.2 (3)
C1—C2—C3—C4	−0.3 (4)	C5—C6—C7—C15	−12.3 (3)
C2—C3—C4—C5	−0.2 (3)	C1—C6—C7—C15	167.1 (2)
C2—C3—C4—N3	179.1 (2)	C13—C8—C9—C10	0.2 (3)
O1—N3—C4—C3	−175.0 (2)	C14—C8—C9—C10	−179.8 (2)
O2—N3—C4—C3	4.4 (3)	C8—C9—C10—C11	0.2 (4)
O1—N3—C4—C5	4.4 (3)	C9—C10—C11—C12	0.2 (4)
O2—N3—C4—C5	−176.2 (2)	C10—C11—C12—C13	−1.1 (4)
C3—C4—C5—C6	0.1 (3)	C11—C12—C13—N1	−177.4 (2)
N3—C4—C5—C6	−179.27 (18)	C11—C12—C13—C8	1.6 (3)
C4—C5—C6—C1	0.6 (3)	N2—N1—C13—C12	−1.0 (3)
C4—C5—C6—C7	−179.92 (18)	N2—N1—C13—C8	−179.92 (17)
C2—C1—C6—C5	−1.2 (3)	C9—C8—C13—C12	−1.1 (3)
C2—C1—C6—C7	179.4 (2)	C14—C8—C13—C12	179.0 (2)
N1—N2—C7—C6	178.38 (16)	C9—C8—C13—N1	177.85 (18)
N1—N2—C7—C15	−1.0 (3)	C14—C8—C13—N1	−2.1 (3)

## References

[bb1] Ahmad, M. S., Pischetsrieder, M. & Ahmed, N. (2007). *Eur. J. Pharmacol.* **561**, 32–38. 10.1016/j.ejphar.2007.01.041 17321518

[bb2] Ahmed, N. (2005). *Diabetes Res. Clin. Pract.* **67**, 3–21. 10.1016/j.diabres.2004.09.004 15620429

[bb3] Bruker (2000). *SADABS*, *SMART* and *SAINT* Bruker AXS Inc., Madison, Wisconsin, USA.

[bb4] Fun, H.-K., Adhikari, A., Patil, P. S., Kalluraya, B. & Chantrapromma, S. (2008). *Acta Cryst.* E**64**, o2286–o2287. 10.1107/S1600536808035939 PMC2959927 21581265

[bb5] Gerdemann, C., Eicken, C. & Krebs, B. (2002). *Acc. Chem. Res.* **35**, 183–191. 10.1021/ar990019a 11900522

[bb6] Khan, K. M., Khan, M., Ali, M., Taha, M., Rasheed, S., Perveen, S. & Choudhary, M. I. (2009). *Bioorg. Med. Chem.* **17**, 7795–7801. 10.1016/j.bmc.2009.09.028 19837595

[bb7] Mallikarjun, S. Y. & Sangamesh, A. P. (1997). *Transition Met. Chem.* **22**, 220–224.

[bb8] Nardelli, M. (1995). *J. Appl. Cryst.* **28**, 659.

[bb9] Sheldrick, G. M. (2008). *Acta Cryst.* A**64**, 112–122. 10.1107/S0108767307043930 18156677

[bb10] Solomon, E. I. & Lowery, M. D. (1993). *Science*, **259**, 1575–1581. 10.1126/science.8384374 8384374

[bb11] Spek, A. L. (2009). *Acta Cryst.* D**65**, 148–155. 10.1107/S090744490804362X PMC2631630 19171970

[bb12] Tezcan, H., Tunc, T., Sahin, E. & Yagbasn, R. (2004). *Anal. Sci.* **20**, 137–138.

